# The role of nutrition in analysis of risk factors and short-term outcomes for late-onset necrotizing enterocolitis among very preterm infants: a nationwide, multicenter study in China

**DOI:** 10.1186/s12887-024-04611-7

**Published:** 2024-03-08

**Authors:** Kun-Yao Hong, Yao Zhu, Fan Wu, Jian Mao, Ling Liu, Rong Zhang, Yan-Mei Chang, Wei Shen, Li-Xia Tang, Xiu-Zhen Ye, Yin-Ping Qiu, Li Ma, Rui Cheng, Hui Wu, Dong-Mei Chen, Ling Chen, Ping Xu, Hua Mei, San-Nan Wang, Fa-Lin Xu, Rong Ju, Zhi Zheng, Xiao-Mei Tong, Xin-Zhu Lin, the Chinese Multicenter EUGR Collaborative Group, Kun Yao Hong, Yao Zhu, Wei Shen, Li-Xia Tang, Zhi Zheng, Xin-Zhu Lin, Fan Wu, Qian-Xin Tian, Qi-Liang Cui, Jian Mao, Yuan Yuan, Ling Ren, Ling Liu, Bi-Zhen Shi, Yu-Mei Wang, Yan-Mei Chang, Jing-Hui Zhang, Xiao-Mei Tong, Rong Zhang, Yan Zhu, Xiu-Zhen Ye, Jing-Jing Zou, Yin-Ping Qiu, Yu-Huai Li, Bao-Yin Zhao, Shu-Hua Liu, Li Ma, Ying Xu, Rui Cheng, Wen-Li Zhou, Hui Wu, Zhi-Yong Liu, Dong-Mei Chen, Jin-Zhi Gao, Jing Liu, Ling Chen, Cong Li, Chun-Yan Yang, Ping Xu, Ya-Yu Zhang, Si-Le Hu, Hua Mei, Zu-Ming Yang, Zong-Tai Feng, San-Nan Wang, Er-Yan Meng, Li-Hong Shang, Fa-Lin Xu, Shaoping Ou, Rong Ju, Gui-Nan Li, Juan Yi, Long Li, Yong-Qiao Liu, Zhe Zhang, Mei-Gui Wu, Fei Bei, Ye Liu, Chun Deng, Hui-Jie Yang, Ping Su, Shi-Feng Chen, Ling-Ying Luo, Lin-Lin Wang, Xiao-Hong Liu, Li-Hua Yan, Li-Jun Wang, Xiao-Kang Wang, Shu-Qun Yu, Qiao-Mian Zhu

**Affiliations:** 1 Department of Neonatology, Women and Children’s Hospital, School of Medicine, Xiamen University, Xiamen, 361003 Fujian China; 2 Xiamen Key Laboratory of Perinatal-Neonatal Infection, Xiamen, China; 3 Department of Neonatology, The Third Affiliated Hospital of Guangzhou Medical University, Guangzhou, China; 4 Department of Pediatrics, Shengjing Hospital of China Medical University, Shenyang, China; 5 Department of Neonatology, Guiyang Maternity and Child Health Hospital, Guiyang Children’s Hospital, Guiyang, China; 6 Department of Neonatology, Children’s Hospital of Fudan University, Shanghai, China; 7 Department of Pediatrics, Peking University Third Hospital, Beijing, 100074 China; 8 Department of Neonatology, Maternal and Children’s Hospital of Guangdong Province, Guangzhou, China; 9 Department of Neonatology, General Hospital of Ningxia Medical University, Yinchuan, China; 10 Department of Neonatology, Children’s Hospital of Hebei Province, Shijiazhuang, China; 11 Department of Neonatology, Children’s Hospital of Nanjing Medical University, Nanjing, China; 12 Department of Neonatology, The First Hospital of Jilin University, Changchun, China; 13 Department of Neonatology, Quanzhou Maternity and Children’s Hospital, Quanzhou, China; 14 Department of Pediatrics, Tongji Hospital, Tongji Medical College, Huazhong University of Science and Technology, Wuhan, China; 15 Department of Neonatology, Liaocheng People’s Hospital, Liaocheng, China; 16 Department of Neonatology, the Affiliate Hospital of Inner Mongolia Medical University, Hohhot, China; 17 Department of Neonatology, Suzhou Municipal Hospital, Suzhou, China; 18 Department of Neonatology, The Third Affiliated Hospital of Zhengzhou University, Zhengzhou, China; 19 Department of Neonatology, School of Medicine, Chengdu Women’ and Children’s Central Hospital, University of Electronic Science and Technology of China, Chengdu, China

**Keywords:** Very preterm infants, Late-onset NEC, Risk factors, Breastfeeding, Extrauterine growth restriction, Late-onset sepsis

## Abstract

**Background:**

Necrotizing enterocolitis (NEC) is a serious gastrointestinal disease, primarily affects preterm newborns and occurs after 7 days of life (late-onset NEC, LO-NEC). Unfortunately, over the past several decades, not much progress has been made in its treatment or prevention. This study aimed to analyze the risk factors for LO-NEC, and the impact of LO-NEC on short-term outcomes in very preterm infants (VPIs) with a focus on nutrition and different onset times.

**Method:**

Clinical data of VPIs were retrospectively collected from 28 hospitals in seven different regions of China from September 2019 to December 2020. A total of 2509 enrolled VPIs were divided into 2 groups: the LO-NEC group and non-LO-NEC group. The LO-NEC group was divided into 2 subgroups based on the onset time: LO-NEC occurring between 8 ~ 14d group and LO-NEC occurring after 14d group. Clinical characteristics, nutritional status, and the short-term clinical outcomes were analyzed and compared among these groups.

**Results:**

Compared with the non-LO-NEC group, the LO-NEC group had a higher proportion of anemia, blood transfusion, and invasive mechanical ventilation (IMV) treatments before NEC; the LO-NEC group infants had a longer fasting time, required longer duration to achieve the target total caloric intake (110 kcal/kg) and regain birthweight, and showed slower weight growth velocity; the cumulative dose of the medium-chain and long-chain triglyceride (MCT/LCT) emulsion intake in the first week after birth was higher and breastfeeding rate was lower. Additionally, similar results including a higher proportion of IMV, lower breastfeeding rate, more MCT/LCT emulsion intake, slower growth velocity were also found in the LO-NEC group occurring between 8 ~ 14d when compared to the LO-NEC group occurring after 14 d (all (*P* < 0.05). After adjustment for the confounding factors, high proportion of breastfeeding were identified as protective factors and long fasting time before NEC were identified as risk factors for LO-NEC; early feeding were identified as protective factors and low gestational age, grade III ~ IV neonatal respiratory distress syndrome (NRDS), high accumulation of the MCT/LCT emulsion in the first week were identified as risk factors for LO-NEC occurring between 8 ~ 14d. Logistic regression analysis showed that LO-NEC was a risk factor for late-onset sepsis, parenteral nutrition-associated cholestasis, metabolic bone disease of prematurity, and extrauterine growth retardation.

**Conclusion:**

Actively preventing premature birth, standardizing the treatment of grade III ~ IV NRDS, and optimizing enteral and parenteral nutrition strategies may help reduce the risk of LO-NEC, especially those occurring between 8 ~ 14d, which may further ameliorate the short-term clinical outcome of VPIs.

**Trial registration:**

ChiCTR1900023418 (26/05/2019).

**Supplementary Information:**

The online version contains supplementary material available at 10.1186/s12887-024-04611-7.

## Background

Necrotizing enterocolitis (NEC) is a serious digestive tract disease which occurs more frequently in very preterm infants (VPIs) during the neonatal period. According to the data of several systematic reviews [1, 2], the incidence of NEC is 7% and the mortality is 20%~30% in premature infants with a birth weight of 500 ~ 1500 g, and there has been no evident improvement in these statistics in recent years. A study enrolling 10,823 VPIs from 57 tertiary neonatal intensive care units (NICUs) of the Chinese Neonatal Network in 2019 showed that the survival rate of VPIs was 95.4%, and the incidence of NEC was 4.9%; notably, the incidence of NEC increased with decreasing gestational age [3]. In 90% cases, NEC occurred in VPIs with a gestational age of < 32 weeks [4]. The risk factors for NEC vary with the time of onset. Short et al. [5]. Proposed an onset time of ≤ 7 days after birth to represent early-onset NEC (EO-NEC) and an onset time of > 7 days after birth as late-onset NEC (LO-NEC). Considering the small amount of enteral nutrition in the early postnatal period, the causes of EO-NEC are mostly related to perinatal factors, such as asphyxia and intrauterine infection. Conversely, LO-NEC is closely related to high-risk factors in the NICU, such as improper feeding, intestinal flora imbalance, long fasting time, and late-onset infection [6]. Most NEC cases occur 13 ~ 23 days after birth, which represents LO-NEC [2]. There is a paucity of data on the related factors of those cases occurring between 8 ~ 14 days after birth, which also belong to LO-NEC. Therefore, analyzing the high-risk factors for LO-NEC with different onset times to prevent the occurrence of NEC has immense guiding significance in clinical practice.

In terms of nutrition, we explored the high-risk factors for LO-NEC and its impact on the short-term clinical outcomes among VPIs through a retrospective analysis of the clinical data from 28 Chinese NICUs in our study. We hope the findings will help further optimize the treatment strategy for premature infants in the NICU, particularly with improving the nutrition management, reducing the incidence of LO-NEC, and providing a theoretical basis for improving the short-term prognosis of VPIs with LO-NEC.

## Methods

### Study design and sample

The data used in this study were sourced from a prospective multicenter study that involved the influencing factors of extrauterine growth restriction (EUGR) in VPIs from different regions of China (clinical trial registry: www.chictr.org.cn; registration number: ChiCTR1900023418; date of first registration: 26/05/2019). The study was approved by the Ethics Committee of the Women and Children’s Hospital, School of Medicine, Xiamen University (No. KY-2019-016). In this study, the clinical data of VPIs in the NICU were collected from 28 tertiary first-class hospitals in 20 provinces of China between September 2019 and December 2020.

#### Inclusion criteria

Infants born with a gestational age (GA) of < 32 weeks or birth weight (BW) of < 1500 g who were admitted to the participating NICUs within 24 h of birth were included.

#### Exclusion criteria

We excluded infants who (1) had a congenital malformation or metabolic disease; (2) died or whose treatment was interrupted, followed by discharge from the hospital because of parents’ wishes or financial constraints; (3) had a hospitalization period of < 2 weeks; (4) had incomplete medical record information; (5) had EO-NEC.

### Study dataset

Depending on whether LO-NEC occurred, VPIs who met the criteria were divided into 2 groups: LO-NEC group and non-LO-NEC group. The LO-NEC group was divided into 2 subgroups based on the onset time: LO-NEC occurring between 8 ~ 14d group and LO-NEC occurring after 14d group. Demographic and clinical data were collected for the following parameters: perinatal information (e.g., GA, BW, the use of antenatal steroids, mode of delivery, the Apgar score at 5 min, and maternal complications during pregnancy); nutrition-related data, including breastfeeding, use of breast milk fortifier, fasting time, weight growth velocity (GV), and parenteral nutrition; and short-term clinical outcomes, such as severe intraventricular hemorrhage (IVH; grade 3 or 4), periventricular leukomalacia (PVL), moderate and severe bronchopulmonary dysplasia (BPD), parenteral nutrition-associated cholestasis (PNAC), and stage 3 through 5 retinopathy of prematurity (ROP) in either eye. Data for diseases and interventions in the LO-NEC group were collected before the occurrence of NEC.

### Study definitions

#### Late-onset NEC

NEC defined as Bell stage II or greater according to established criteria [7]. The onset time of > 7 days after birth was defined as LO-NEC [5]. In this study, after removing excluded cases (as described above), infants diagnosed as having confirmed NEC (≥ stage II) with an onset time of > 7 days after birth were included in the LO-NEC group, and the remaining infants were enrolled in the non-LO-NEC group.

#### Definitions related to nutrition management

(1) Early feeding was defined as starting feeding within 24 h of birth. (2) High proportion of breastfeeding was defined as breastfeeding accounting for more than 50% of the total enteral feeding during hospitalization. (3) The average weight GV (after regaining BW, g/kg/day) = [1000 × ln(Wn/W1)]/(Dn - D1). Wn: The weight of the infant at the day of discharge (g); W1: The weight of the infant on the first day after delivery (g); Dn: Length of hospitalization in days (d); and D1: the length of regaining BW in days (d) [8].

#### Definitions related to diseases (including short-term outcomes)

(1) Hemodynamically significant patent ductus arteriosus (hsPDA) was defined as a patent ductus arteriosus catheter diameter of > 1.5 mm, left atrial diameter/aortic diameter of ≥ 1.4, or left ventricular end-diastolic diameter/aortic diameter of ≥ 2.1 accompanied by one of the following clinical manifestations: heart murmur, tachycardia (sustained ≥ 160 beats/min), increased respiration, increased pulse pressure (*>* 25 mmHg), hypotension, flushing, or cardiac dilation [9]. (2) The diagnostic criteria of early-onset sepsis (EOS) and late-onset sepsis (LOS; including clinical and diagnosis criteria) were based on the Expert Consensus on the Diagnosis and Treatment of Neonatal Sepsis (2019 Edition) [10]. (3) The EUGR evaluation criteria refer to the Fenton growth curve published in 2013 [11]. ① The evaluation criteria for percentile were as follows: VPI with a weight below the 10th percentile at the time of discharge or 36 weeks of corrected gestational age; ② A change in the *z*-score (Δ*z* value) of weight by more than 1 between two points (discharge/36 weeks of corrected gestational age and birth). (4) Moderate and severe BPD, severe IVH (grade 3 or 4), and stage 3–5 ROP in either eye were diagnosed as per standard definitions and the published literature [12–14]. (5) Finally, diagnoses of grade III–IV neonatal respiratory distress syndrome (NRDS), PNAC, PVL, metabolic bone disease of prematurity (MBDP), and anemia were established by referring to Practical Neonatology (5th edition) [15].

### Statistical methods

The counting data rate (%) indicated that a comparison between groups was performed using *χ*^*2*^ test. The Kolmogorov–Smirnov test was used to evaluate whether the measurement data conformed to the normal distribution. Normally distributed measurement data were expressed as ± *s*, and the two independent samples *t*-test was used for between-group comparisons, whereas non-normally distributed measurement data were expressed as *M* (*Q1, Q3*). The rank-sum test was used for between-group comparisons. Multivariate analysis was performed using binary/triple logistic regression analysis and linear regression analysis. All statistical analyses were conducted using a software program (SPSS, version 26.0; IBM, Armonk, NY, USA), with statistical significance evaluated using two-sided *P* values at the 5% testing level.

## Results

### General information

**Fig. 1:**
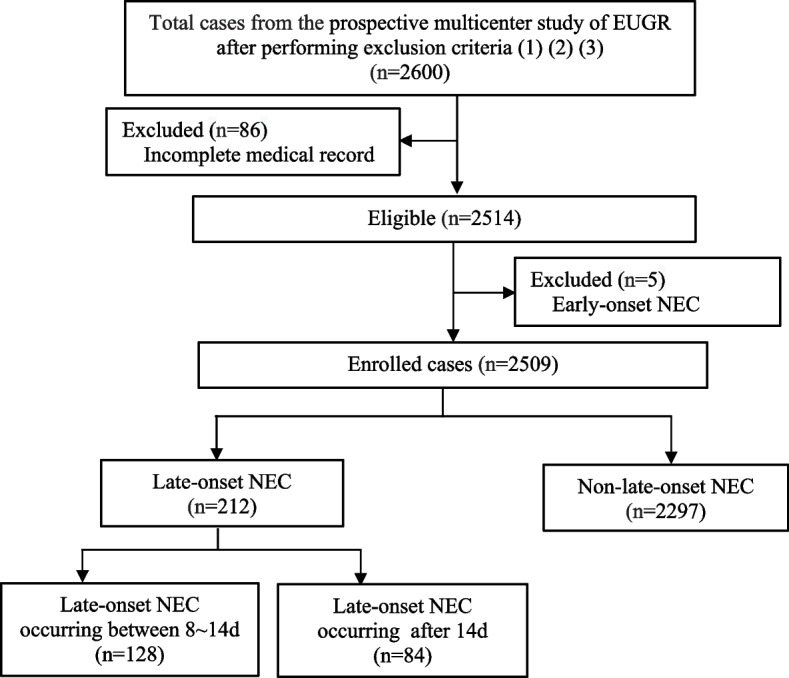
Subject enrollment and selection flow chart

**Fig. 2:**
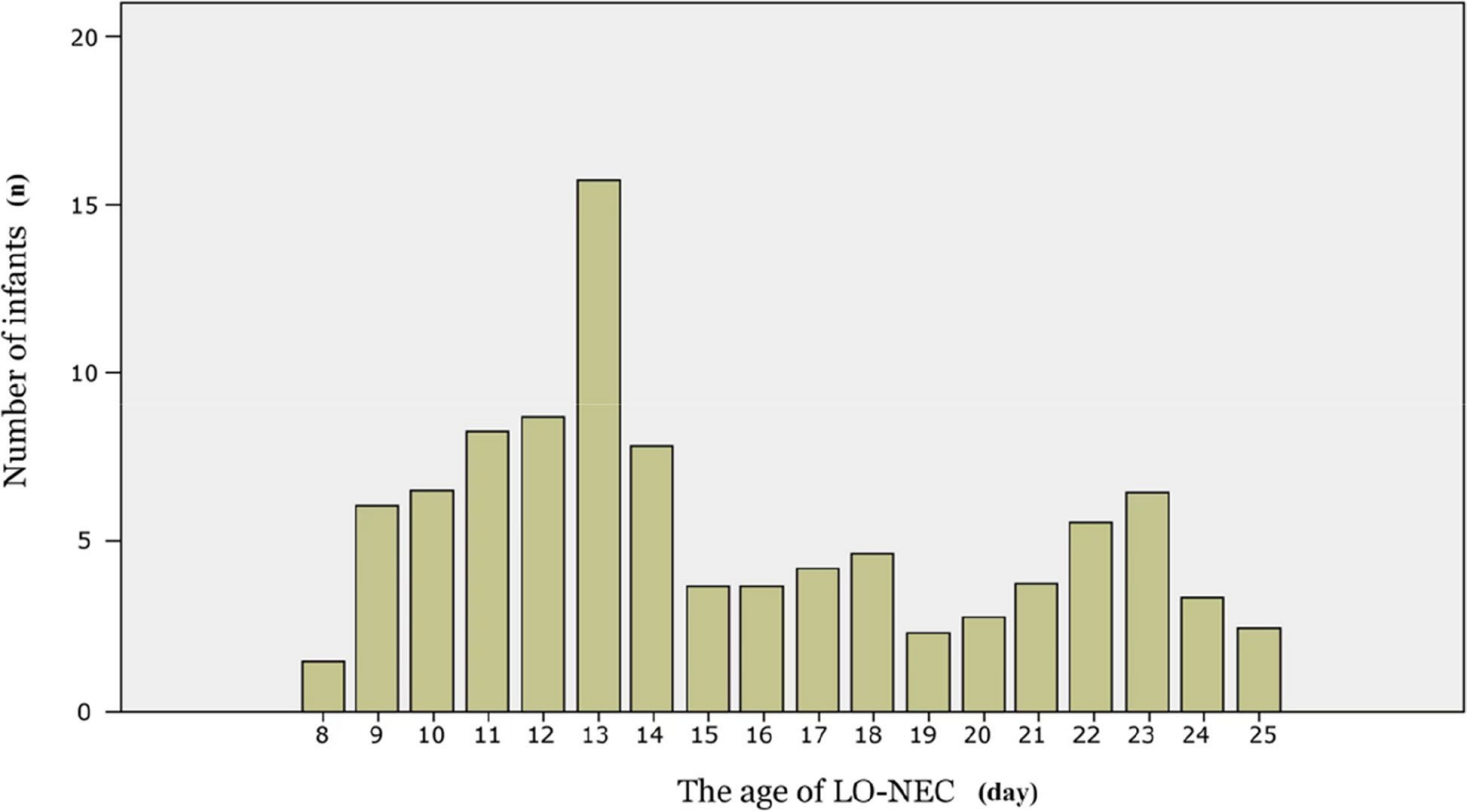
Numbers of participants according to age of late-onset necrotizing enterocolitis

A total of 2600 preterm infants with a GA of < 32 weeks were admitted during the study period. After excluding 86 cases with incomplete medical information and 5 EO-NEC cases, the remaining 2509 infants enrolled in the analysis included 212 cases (8.4%) in the LO-NEC group and 2297 cases (91.6%) in the non-LO-NEC group (Fig. 1). Among LO-NEC VPIs, 47 cases were received surgical treatment, and 165 cases were received conservative treatment. Clinical characteristics of the LO-NEC VPIs with these two treatment methods were presented in Supplementary Table 1. The median age of VPIs in the LO-NEC group was 14 days (11,19); 60.4% of them were 8 ~ 14 days old, and 39.6% were > 14 days old (Fig. 2).

### Comparison of perinatal characteristics

Compared with infants in the non-LO-NEC group, those in the LO-NEC occurring between 8 ~ 14d group had a lower GA (29.8 weeks vs. 30.1 weeks) and those in the LO-NEC occurring after 14 d group had a lower BW (1260.0 g vs. 1340.0 g); furthermore, the LO-NEC group had a higher proportion of anemia, blood transfusion, and invasive mechanical ventilation (IMV) treatments before NEC. The 3 groups did not significantly differ in other perinatal factors, such as mode of delivery, male:female ratio, multiple births, percentage of small for gestational age (SGA) infants, full-course antenatal steroids, maternal complications (e.g., gestational diabetes mellitus and hypertensive disorder complicating the pregnancy), and underlying disease (e.g., EOS, grade III ~ IV NRDS, and hsPDA) (all *P* > 0.05; Table 1).

**Table 1:** Perinatal and clinical data of VPIs among the three groups

Variable	LO-NEC		non-LO-NEC	χ2/Z	*P*
	LO-NEC occurring between 8 ~ 14d	LO-NEC occurring after 14d			
	(*n* = 128)	(*n* = 84)	(*n* = 2297)		
GA, [*M*(*Q1,Q3*)], weeks	29.8 (28.5,30.7)^a^	30.0 (28.8,30.9)	30.1 (28.9,31.1)	3.670	0.026
BW, [*M*(*Q1,Q3*)], grams	1285 (1120,1490)	1260 (1092,1450)^a^	1340 (1130,1550)	6.841	0.001
Cesarean section, *n*(%)	79 (61.7)	52 (62.0)	1402 (61.0)	0.047	0.977
Multiple gestation, *n*(%)	44 (34.3)	19(22.6)	758 (33.0)	4.134	0.127
Male gender, *n*(%)	67 (52.3)	37(44.0)	1271 (55.3)	4.496	0.106
Gestational diabetes mellitus, *n*(%)	26 (20.3)	16 (19.0)	389 (16.9)	1.186	0.553
HDCP, *n*(%)	24 (18.7)	23(27.3)	463 (20.2)	2.819	0.244
Full-course antenatal steroids^c^, *n*(%)	64 (50.0)	41 (48.8)	1118 (48.7)	0.086	0.958
Fetal distress, *n*(%)	12 (9.3)	4(4.7)	205 (8.9)	1.802	0.406
Apgar score ≤ 7 at 5 min, *n*(%)	16 (12.5)	15(17.8)	277 (12.1)	2.536	0.281
SGA, *n*(%)	8 (6.3)	5 (5.9)	119 (5.2)	0.361	0.835
Anemia^d^, *n*(%)	121 (94.5)^a^	78 (92.8)^a^	1912 (83.2)	16.535	< 0.001
Grade III-IV NRDS^e^, *n*(%)	24 (18.8)	14 (16.7)	336 (14.6)	1.837	0.399
EOS, *n*(%)	20 (15.6)	13 (15.5)	336 (14.6)	0.137	0.934
hsPDA, *n*(%)	23 (17.9)	16 (19.0)	392 (17.1)	0.283	0.868
Blood transfusion^d^, *n*(%)	99 (77.3)^a^	68 (80.9)^a^	1319 (57.4)	36.912	< 0.001
Antibiotics^d^, *n*(%)	126 (98.4)	81 (96.4)	2185 (95.2)	3.096	0.213
Duration of antibiotics^d^, [*M*(*Q1,Q3*)], days	19.0 (13.0, 28.0)^a^	27.0 (17.0, 34.0)^a,b^	11.0 (8.0, 19.0)	79.752	< 0.001
IMV^d^, *n*(%)	79 (61.7)^a,b^	48 (57.1)	1101 (47.9)	11.560	0.003
Postnatal corticosteroid use^d^, *n*(%)	23 (18.0)	15 (17.8)	315 (13.7)	3.300	0.770

*GA* Gestational age, *BW* Birth weight, *HDCP* Hypertensive disorder complicating pregnancy, *SGA* Small for gestational age, *NRDS* Neonatal respiratory distress syndrome, *EOS* Early-onset sepsis, *hsPDA* Hemodynamically significant patent ductus arteriosus, *IMV* Invasive mechanical ventilation

^a^Signifcantly diferent between the non-LO-NEC group and LO-NEC group

^b^Signifcantly diferent between the LO-NEC occurring between 8 ~ 14d group and LO-NEC occurring after 14d group

^c^A complete course of antenatal betamethasone consisted of two intramuscular 12-mg doses that were administered to mothers 24 h apart

^d^Data for diseases and interventions in the LO-NEC group were collected before the occurrence of NEC

^e^All Grade III-IV NRDS patients have received IMV treatment

### Comparison of nutritional status

Compared with infants in the non-LO-NEC group, those in the LO-NEC group took longer to reach 110 kcal/(kg·d) total caloric intake had a longer fasting time; those in the LO-NEC occurring between 8 ~ 14d group took longer to regain BW (10.5 days vs. 9.0 days; all *P* < 0.05). Furthermore, infants in the LO-NEC occurring between 8 ~ 14d group had a lower breastfeeding rate, lower GV, and higher cumulative dose of medium-chain and long- chain triglyceride (MCT/LCT) emulsion in the first week after birth than those in the LO-NEC occurring after 14 d group and the non-LO-NEC group. The 3 groups did not significantly differ in terms of other nutritional factors, such as breast milk volume on addition of human milk fortifier, days for full human milk fortifier (HMF), cumulative dose of amino acids, cumulative dose of SMOF® (soybean oil, medium-chain triglycerides, olive oil, and fish oil) lipid emulsion, and cumulative caloric intake in the first week after birth (*P* > 0.05; Table 2).

**Table 2:** Nutrition-related characteristics of VPIs among the three groups

Variable	LO-NEC		non-LO-NEC	χ2/Z	* P*
	LO-NEC occurring between 8 ~ 14d	LO-NEC occurring after 14d			
	(*n* = 128)	(*n* = 84)	(*n* = 2297)		
Early feeding, *n*(%)	65 (50.8)	47 (56.0)	1217 (53.0)	0.546	0.761
Fasting time^c^, [*M*(*Q1,Q3*)], days	7.8 (4.0,10.0)^a^	6.7 (4.5,8.0)^a^	1.5 (0.7,4.0)	187.127	< 0.001
High proportion of breastfeeding, *n*(%)	27 (21.1)^a,b^	33 (39.3)	1072 (46.7)	25.437	< 0.001
Breast milk volume on addition of HMF, [M ( Q1,Q3 )], ml/kg/day	97.8 (91.9,120.0)	100.0 (90.2,114.2)	102.9 (89.8,128.0)	1.748	0.174
Days for full fortification, [*M* (*Q1,Q3*)], days	7.5 (3.0,13.5)	9.0(8.0,15.0)	7.0 (4.0,18.0)	0.143	0.886
Cumulative dose of amino acids during W1, [M ( Q1,Q3 )], g/kg	16.1 (13.4,18.4)	16.4 (14.1,19.5)	16.0 (13.3,18.4)	0.874	0.417
Cumulative dose of MCT/LCT emulsion during W1, [*M* (*Q1,Q3* )], g/kg	13.8 (10.2,15.9)^a,b^	12.6 (9.5,13.8)	12.2 (9.7,14.7)	28.914	< 0.001
Cumulative dose of SMOF lipid emulsion during W1, [*M* (*Q1,Q3* )], g/kg	14.0 (12.5,14.2)	14.2 (12.2,15.6)	13.6 (10.5,16.0)	0.117	0.889
Cumulative calories during W1, [*M* (*Q1,Q3* )], kcal	493.0 (421.3,569.9)	477.4 (420.1,550.1)	497.5 (422.2,564.1)	0.757	0.469
Age at reaching the target total calorie intake (110 kcal/kg), [ M ( Q1,Q3 )], days	10.5 (8.0,12.0)^a^	10.0 (7.0,17.0)^a^	9.0 (7.0,14.0)	3.093	0.046
Days to regain BW, [*M* (*Q1,Q3* )], days	10.5 (8.0,12.5)^a^	9.5 (6.5,11.5)	9.0 (7.0,12.0)	7.73	< 0.001
Weight growth velocity, [*M* (*Q1,Q3* )], g/kg/day	13.8 (10.6,16.0)^a,b^	15.2 (13.5,18.8)	16.1 (12.9,18.3)	5.457	0.004

*HMF* Human milk fortifier, *W1* the first week after birth, *MCT/LCT* Medium-chain and long-chain triglyceride, *BW* Birth weight

^a^Signifcantly diferent between the non-LO-NEC group and LO-NEC group

^b^Signifcantly diferent between the LO-NEC occurring between 8 ~ 14d group and LO-NEC occurring after 14d group

^c^Data for fasting time in the LO-NEC group were collected before the occurrence of NEC

### Multivariate logistic regression analysis of the risk factors for LO-NEC in VPIs

Although univariate analysis revealed no significant differences in the incidence of early feeding among 3 groups (*P* = 0.762), it was still included in the multivariate regression analysis considering its protective effects against NEC [16]. After adjusting for factors that may affect LO-NEC, multivariate analysis showed that high proportion of breastfeeding (a*OR*: 0.25, 95%*CI*: 0.10 ~ 0.78; a*OR*: 0.53, 95%*CI*: 0.08 ~ 0.89) were identified as protective factors and long fasting time before NEC (a*β*: -4.65, 95%*CI*: -6.02~-2.14; a*β*: -3.32, 95%*CI*: -5.18~-2.07) were identified as risk factors for LO-NEC; early feeding (a*OR*: 0.42, 95%*CI*: 0.14 ~ 0.84; a*OR*: 0.28, 95%*CI*: 0.19 ~ 0.77) were identified as protective factors and low gestational age (a*β*: -1.98, 95%*CI*: -5.64~-0.45), grade III ~ IV neonatal respiratory distress syndrome (NRDS) (a*OR*: 3.32, 95%*CI*: 1.76 ~ 6.45), high accumulation of the MCT/LCT emulsion in the first week (a*β*: -3.44, 95%*CI*: -7.19~-1.16; a*β*: -3.59, 95%*CI*: -6.45~-2.76) were identified as risk factors for LO-NEC occurring between 8 ~ 14d (Table 3).

**Table 3:** Multivariate logistic regression analysis of the risk factors for the occurrence of LO-NEC in VPIs

Variables	LO-NEC onset			a*β/OR* ^a^ (95% *CI*) 8 ~ 14d vs. 0d	a*β/OR* ^a^ (95% *CI*) > 14d vs. 0d	a*β/OR* ^a^ (95% *CI*) 8 ~ 14d vs. > 14d
	LO-NEC occurring between 8 ~ 14d	LO-NEC occurring after 14d	non-LO-NEC			
	(*n* = 128)	(*n* = 84)	(*n* = 2297)			
Early feeding	65 (50.8)	47 (56.0)	1217 (53.0)	0.42 (0.14 ~ 0.84)	0.94 (0.47 ~ 2.89)	0.28 (0.19 ~ 0.77)
High proportion of breastfeeding	27 (21.1)	33 (39.3)	1072 (46.7)	0.25 (0.10 ~ 0.78)	0.53 (0.08 ~ 0.89)	0.37 (0.15 ~ 0.82)
Fasting time	7.8 (4.0,10.0)	6.7 (4.5,8.0)	1.5 (0.7,4.0)	-4.65 (− 6.02 ~ − 2.14)	-3.32 (− 5.18 ~ − 2.07)	-1.12 (− 7.88 ~ 2.55)
Cumulative dose of MCT/LCT emulsion during W1	13.8 (10.2,15.9)	12.6 (9.5,13.8)	12.2 (9.7,14.7)	-3.44 (− 7.19 ~ − 1.16)	-2.07 (− 8.43 ~ 1.97)	-3.59 (− 6.45 ~ − 2.76)
Gestational age	29.8 (28.5,30.7)	30.0 (28.8,30.9)	30.1 (28.9,31.1)	-1.98 (− 5.64 ~ − 0.45)	-1.34 (− 6.78 ~ 4.96)	-1.47 (− 5.19 ~ 3.84)
Grade III-IV NRDS	24 (18.8)	14 (16.7)	336 (14.6)	3.32 (1.76 ~ 6.45)	2.11 (0.75 ~ 5.87)	2.43 (0.68 ~ 7.54)

MCT/LCT Medium-chain and long-chain triglyceride, NRDS Neonatal respiratory distress syndrome, HMF Human milk fortifier, W1 the first week after birth, BW Birth weight, IMV Invasive mechanical ventilation, hsPDA Hemodynamically significant patent ductus arteriosus, EOS Early-onset sepsis

^a^Adjusted for the amount of HMF, days for full fortification, cumulative calories during W1, days to regain BW, IMV, Apgar score ≤ 7 at 5 min, antenatal steroids, hsPDA, EOS, anemia requiring blood transfusion

### Multivariate logistic regression analysis of LO-NEC for short-term outcomes in VPIs

Univariate analysis found that the incidence of LOS, MBDP, PNAC, and EUGR was statistically significantly different between the two groups (all *P* < 0.05; Table 4). After adjusting for confounding factors that may affect the clinical outcomes, multivariate regression analysis showed that the risk of LOS was 1.5 times higher in the LO-NEC group than in the non-LO-NEC group (a*OR* = 1.527, 95% *CI*: 1.049 ~ 2.221, *P* < 0.001), the risk of MBDP was 2.6 times higher in the LO-NEC group than in the non-LO-NEC group (a*OR* = 2.613, 95% *CI*: 1.363 ~ 5.008, *P* = 0.002), the risk of PNAC was 2.3 times higher in the LO-NEC group than in the non-LO-NEC group (a*OR* = 2.350, 95% *CI*: 1.616 ~ 3.416, *P* < 0.001), and the risk of EUGR was 1.6 times higher in the LO-NEC group than in the non-LO-NEC group (a*OR* = 1.613, 95% *CI*: 1.114 ~ 2.337, *P* < 0.001; Table 5).

**Table 4:** Short-term outcomes of VPIs in the LO-NEC and non-LO-NEC groups

Outcomes	LO-NEC (*n* = 212)	non-LO-NEC (*n* = 2297)	χ2/Z	* P*
LOS, *n*(%)	53 (25.0)	270(11.8)	30.359	< 0.001
Moderate and severe BPD, *n*(%)	37 (17.5)	363(15.8)	0.394	0.530
ROP requiring intervention, *n*(%)	9 (4.2)	70 (3.0)	0.913	0.339
IVH (grade 3 or 4), *n*(%)	2 (0.9)	48 (2.1)	1.306	0.253
PVL, *n*(%)	9 (4.2)	84 (3.7)	0.188	0.664
MBDP, *n*(%)	13 (6.1)	57 (2.5)	9.537	0.002
PNAC, *n*(%)	53 (25.0)	205 (8.9)	54.365	< 0.001
EUGR, *n*(%)	134 (63.2)	1046 (45.5)	25.132	< 0.001

*LOS* Late-onset sepsis, *BPD* Bronchopulmonary dysplasia, *ROP* Retinopathy of prematurity, *IVH* Intraventricular hemorrhage, *PVL* Periventricular leukomalacia, *MBDP* Metabolic bone disease of prematurity, *PNAC* Parenteral nutrition associated cholestasis, *EUGR* Extrauterine growth restriction

**Table 5:** Multivariate logistic regression analysis of LO-NEC for short-term outcomes in VPIs

Outcomes	LO-NEC (*n* = 212)	non-LO-NEC (*n* = 2297)	OR^a^ (95% CI)	* P*
LOS	53 (25.0)	270 (11.8)	1.527 (1.049 ~ 2.221)	0.027
Moderate and severe BPD	37 (17.5)	363 (15.8)	0.654 (0.420 ~ 1.020)	0.061
ROP requiring intervention	9 (4.2)	70 (3.0)	1.122 (0.517 ~ 2.432)	0.771
IVH (grade 3 or 4)	2 (0.9)	48 (2.1)	0.405 (0.096 ~ 1.716)	0.220
PVL	9 (4.2)	84 (3.7)	1.200 (0.579 ~ 2.488)	0.624
MBDP	13 (6.1)	57 (2.5)	2.613 (1.363 ~ 5.008)	0.004
PNAC	53 (25.0)	205 (8.9)	2.350 (1.616 ~ 3.416)	< 0.001
EUGR	135 (63.7)	1050 (45.7)	1.613 (1.114 ~ 2.337)	0.011

*LOS* Late-onset sepsis, *BPD* Bronchopulmonary dysplasia, *ROP* Retinopathy of prematurity, *IVH* Intraventricular hemorrhage, *PVL* Periventricular leukomalacia, *MBDP* Metabolic bone disease of prematurity, *PNAC* Parenteral nutrition associated cholestasis, *EUGR* Extrauterine growth restriction, *SGA* Small for gestational age, *PN* Parenteral nutrition, *NRDS* Neonatal respiratory distress syndrome, *IMV* Invasive mechanical ventilation

^a^Adjusted for gestational age, birth weight, SGA, antenatal steroids, breastfeeding, duration of PN, grade III-IV NRDS, anemia,IMV, antibiotics

## Discussion

LO-NEC is one of the main causes of death in premature infants one week after birth [17]. The early clinical manifestations and X-ray imaging results of NEC are often nonspecific, and the progress is rapid. By the time its diagnosis is established, the condition is often serious and generally presents with intestinal necrosis and perforation, which require surgical treatment. Many of the survivors present with short bowel syndrome, which can widely be complicated with intestinal stenosis, PNAC, EUGR, and other short-term adverse prognoses and even with neurocognitive dysfunction in the long term. Therefore, the prevention of this disease is the most critical, and to this end, it is necessary to recognize the high-risk factors for NEC early and intervene promptly, which can greatly improve the clinical outcome of premature infants.

At present, LO-NEC is considered to result from multiple factors which are mainly related to an altered intestinal barrier immune response to feeding and the developing microbiome in premature infants; LO-NEC development can also be attributed to hypoxia, hemodynamic instability, and infection [1, 18]. In our study, compared to the non-LO-NEC group, the LO-NEC group had a higher incidence of anemia, although there was no difference after adjustment. Anemia can impair splanchnic perfusion, resulting in tissue hypoxia. Anemia can also impair the normal maturation of vascular autoregulation in the premature intestine, predisposing to ischemic injury, and possibly, NEC [19]. Therefore, the early prevention and treatment of anemia is encouraged in the NICU. Potential means of preventing anemia include delayed cord clamping and methods for limiting phlebotomy losses.

This study confirmed that low GA and grade III ~ IV NRDS were high-risk factors for LO-NEC occurring between 8 ~ 14d. A lower GA with a higher incidence of grade III ~ IV NRDS is associated with intestinal immaturity and vulnerability to various pathological factors, consequently leading to NEC [20]. In VPIs with grade III ~ IV NRDS, immature lungs have damaged gas exchange function and intestinal tissue oxygenation disorder, which lead to NEC. In our study, all grade III-IV NRDS patients have received IMV treatment. Compared to the LO-NEC group occurring after 14 d, higher proportion of grade III ~ IV NRDS and longer IMV duration were found in the LO-NEC group occurring between 8 ~ 14d. It has been hypothesized that indwelling endotracheal tube may compromise the infants’ mucosal barriers increasing the risk of infection [21]. Moreover, variations in intrathoracic pressure during IMV had a major impact and affect systemic venous return, right ventricular preload, left ventricular preload, right ventricular afterload, left ventricular afterload and myocardial contracility, which may affect blood supply to the intestine and consequently increase the risk of NEC [22].

A long fasting time may lead to intestinal mucosal atrophy, intestinal dysfunction, abnormal intestinal flora colonization, and excessive intestinal inflammatory response in premature infants, which are high-risk factors for NEC [23]. Notably, enteral feeding can prevent intestinal atrophy, enhance mucosal adaptability, and stimulate intestinal movement and growth. This study shows that early feeding is a protective factor against LO-NEC occurring between 8 ~ 14d. A prospective randomized controlled trial showed that VPIs given trophic feeding within 24 h of birth had faster body weight growth and that this approach helped prevent NEC [16]. In agreement with previous studies, the present study also confirmed that breastfeeding was a protective factor for LO-NEC. The protective effect of breastfeeding on NEC reportedly has a dose–effect relationship. Lapidaire et al. confirmed that every 10% increase in breast milk intake was associated with a ~ 12% reduction in the incidence of NEC [24]. Miller et al. [25]. also reported a more evident protective effect when the intake of breast milk was ≥ 50% of the total intake. However, the overall breastfeeding ratio in this study was not high because most NICUs in China did not receive breast milk during the COVID-19 pandemic; this may also explain the incidence of LO-NEC being slightly higher in this study (8.4%) than in other studies (7.0%) [2].

The results of this study showed that a higher cumulative dose of the MCT/LCT emulsion in the first week was a risk factor for LO-NEC occurring between 8 ~ 14d; however, the cumulative dose of SMOF did not statistically significantly differ between the two groups. Some investigators have pointed out that the use of lipid emulsion impairs monocyte, lymphocyte, and neutrophil functions, and these changes seemed to be related to the quantity and rate of lipid administration; conversely, the use of n-3 fatty acids (represented by SMOF) produces less immunosuppressive eicosanoids [26]. A major finding of a previous study was that compared to the standard dose of intravenous lipid emulsion (starting from 0.5 g/kg/day^−1^ and gradually increased by 1 g/kg/day to a maximum of 3.5 g/kg/day), a restricted dose of intravenous lipid emulsion (1 g/kg/day) significantly increased the rate of bacterial clearance in preterm infants with blood stream infections [27]. A significant increase in the levels of n-6 long-chain polyunsaturated fatty acids (LCPUFAs), proinflammatory lipid metabolites, such as arachidonic acid-containing glycerophospholipids, and eicosanoids, such as prostaglandin A2 (PGA2), was observed in the intestinal tissue of NEC pigs [28]. The MCT/LCT emulsion contains a high proportion of ω-6 LCPUFAs (ω-6:ω-3 = 7:1). ω-6 Arachidonic acid (AA), a metabolite of LCPUFA, is a precursor of inflammatory mediators. It can catalyze the formation of PG and TBX *via* cyclooxygenase, reduce and weaken the phagocytosis of neutrophils, promote oxidative stress injury, increase the production of inflammatory mediators, and damage the vascular endothelial system and immunosuppression [29], thus resulting in an increased risk of NEC. In addition, ω-6 LCPUFAs metabolize to produce a strong vasoconstrictor, TXA2, whereas the vasodilator PGI2 is significantly reduced; these changes constrict the blood vessels and form thrombi in severe cases [25, 29], thus affecting intestinal blood flow and inducing NEC. This study also confirmed that early feeding was a protective factor for LO-NEC occurring between 8 ~ 14d, whereas a long fasting time was a risk factor for LO-NEC. A long fasting time and late feeding of VPIs will inevitably increase the cumulative dose of the MCT/LCT emulsion ingested intravenously in the first week, which may further increase the risk of NEC. Therefore, enteral nutrition should be started as soon as possible to minimize the dosage of the MCT/LCT emulsion in the first week. Notably, giving SMOF lipid emulsion with less ω-6 LCPUFAs may be beneficial to reduce the incidence of LO-NEC. Similarly, regarding nutrition aspects, lower early feeding rate and breastfeeding rate, more MCT/LCT emulsion intake, slower growth velocity were found in the LO-NEC group occurring between 8 ~ 14d when compared to the LO-NEC group occurring after 14 d. These results may suggest that nutrition plays a role in affecting the timing of NEC occurrence, which is worth further exploration.

NEC occurring in VPIs may increase the risk of short-term adverse outcomes. Shah et al. [30]. reported that NEC was associated with an increased risk of ROP, BPD, and PVL. Compared with the non-LO-NEC group, our study found that the incidence of EUGR, LOS, PNAC, and MBDP in the LO-NEC group was 1.6, 1.5, 2.4, and 2.6 times higher, respectively. A foreign study has pointed out that more than half of NEC premature infants have EUGR [31]. A recent domestic study also confirmed that the risk of development of EUGR was 5 times higher in very low-birth-weight infants with confirmed NEC, regardless of whether it was treated surgically [32]. The reasons were considered to be related to delayed feeding, longer fasting time, slower GV, longer time to regain BW, longer time to reach total enteral nutrition, and longer time to reach a total caloric intake of 110 kcal/kg/d in the LO-NEC group. Less nutrient intake and malabsorption caused by short bowel syndrome or intestinal stenosis after intestinal resection also increase the incidence of EUGR [33]. Due to NEC, the parenteral nutrition (PN) time is prolonged with decreased intestinal peristalsis, which may lead to intestinal flora translocation through the damaged intestinal wall, causing bacteria and toxins to invade, which leads to an increased risk of LOS. In addition, endotoxin invades the portal venous system, leading to the activation of liver Kupffer cells in the early stage, which results in hepatocyte damage and cholestasis [34]. A study using a mouse model found that when there was intestinal injury and the PN time was prolonged, which lead to hepatocyte injury and PNAC occurrence, neither the intestinal injury nor the PN prolongation alone was sufficient to trigger the above results, suggesting that the combined effect of intestinal injury and PN prolongation led to the occurrence of PNAC [35]. NEC and PNAC in premature infants delayed establishment of enteral nutrition, and a PN time of > 4 weeks are reportedly high-risk factors for MBDP [36].

This study had several limitations. First, this study lacks information regarding some important aspects, such as chorioamnionitis during pregnancy, delayed cord clamping, hypoxic events, impaired placental function, and the use of probiotics. These confounding factors may have influenced the clinical outcomes. Second, the data presented here come from a prospective multicenter study on factors influencing VPIs-EUGR in China, which excluded patients who died at the initial stage and during hospitalization. Therefore, the effect of NEC on death as a clinical outcome was not studied. Finally, nutrition management strategies of each center are slightly different, particularly enteral nutrition, and therefore, the research results may be biased.

## Conclusion

The results of this study showed that long fasting time, high cumulative dose of the MCT/LCT emulsion in the first week, and low GA with grade III–IV NRDS were the risk factors for LO-NEC in VPIs, especially those occurring between 8 ~ 14d; conversely, early feeding and breastfeeding were protective factors. Therefore, it is necessary to actively prevent premature birth, standardize the treatment of grade III ~ IV NRDS and optimize enteral and PN strategies, which may help reduce the incidence of LO-NEC and may further reduce the risk of clinical adverse outcomes, such as EUGR, LOS, PNAC, and MBDP.

### Supplementary Information

**Supplementary material 1.**

## Data Availability

The datasets generated and analyzed during the current study are available from the corresponding author on reasonable request.

## Abbreviations

NEC: Necrotizing enterocolitis
EO-NEC: Early-onset NEC
LO-NEC: Late-onset NEC
VPIs: Very preterm infants
MCT/LCT: Medium-chain and long-chain triglyceride
NRDS: Neonatal respiratory distress syndrome
NICUs: Neonatal intensive care units
EUGR: Extrauterine growth restriction
GA: Gestational age
BW: Birth weight
GV: Growth velocity
IVH: Intraventricular hemorrhage
PVL: Periventricular leukomalacia
BPD: Bronchopulmonary dysplasia
PNAC: Parenteral nutrition-associated cholestasis
ROP: Retinopathy of prematurity
hsPDA: Hemodynamically significant patent ductus arteriosus
EOS: Early-onset sepsis
LOS: Late-onset sepsis
PMA: Postmenstrual age
NRDS: Neonatal respiratory distress syndrome
MBDP: Metabolic bone disease of prematurity
SGA: Small for gestational age
HMF: Human milk fortifier
SMOF: Soybean oil, medium-chain triglycerides, olive oil, and fish oil
LCPUFAs: Long-chain polyunsaturated fatty acids
PGA2: Prostaglandin A2
AA: Arachidonic acid
PG: Prostaglandin
TBX: Thromboxane
TXA2: Thromboxane A2
PGI2: Prostaglandin-I-2
PN: Parenteral nutrition
HDCP: Hypertensive disorder complicating pregnancy
IMV: Invasive mechanical ventilation
