# Supplementary material for: The role of nutrition in analysis of risk factors and short-term outcomes for late-onset necrotizing enterocolitis among very preterm infants: a nationwide, multicenter study in China
Source: BMC Pediatr. 2024 Mar 8;24:172. doi: 10.1186/s12887-024-04611-7 (PMC10921728; doi:10.1186/s12887-024-04611-7)
Supplement: Supplementary file 1 — Supplementary material 1. [file 12887_2024_4611_MOESM1_ESM.docx]

| **Supplementary Table 1 Clinical characteristics of the LO-NEC VPIs with different treatment methods** | | |
| --- | --- | --- |
| Variables | LO-NEC with surgical treatment  (n=47) | LO-NEC with conservative treatment  (n=165) |
| Perinatal characteristics | | |
| GA, [*M*(*Q1,Q3*)], weeks | 29.9 (28.5,30.6) | 30.0 (28.4,31.0) |
| BW, [*M*(*Q1,Q3*)], grams | 1280 (1120,1485) | 1268 (1090,1478) |
| Cesarean section, *n*(%) | 29 (61.7) | 102 (61.8) |
| Gestational diabetes mellitus, *n*(%) | 10 (21.3) | 32 (19.4) |
| Full-course antenatal steroids^a^, *n*(%) | 23 (48.9) | 82 (49.7) |
| Apgar score ≤7 at 5 min, *n*(%) | 7 (14.9) | 24 (14.5) |
| SGA, *n*(%) | 3 (6.4) | 10 (6.0) |
| Clinical diseases and interventions | | |
| Age of LO-NEC onset, [*M* (*Q1,Q3*)], days | 15.0 (12.0,21.0) | 12.0 (9.0,18.0) |
| Grade III-IV NRDS, *n*(%) | 9 (19.1) | 29 (17.6) |
| EOS, *n*(%) | 7 (14.9) | 26 (15.8) |
| hsPDA, *n*(%) | 8 (17.0) | 31 (18.8) |
| Anemia^b^, *n*(%) | 45 (95.7) | 154 (93.3) |
| Blood transfusion^b^, *n*(%) | 40 (85.1) | 127 (77.0) |
| Antibiotics^b^, *n*(%) | 47 (100.0) | 160 (96.9) |
| IMV^b^, *n*(%) | 33 (70.2) | 94 (56.9) |
| Postnatal corticosteroid use^b^, *n*(%) | 8 (17.0) | 30 (18.1) |
| Nutrition-related characteristics | | |
| Early feeding, *n*(%) | 23 (48.9) | 89 (53.9) |
| Fasting time^b^, [*M*(*Q1,Q3*)], days | 8.0 (4.5,11.0) | 6.5 (4.0,7.5) |
| High proportion of breastfeeding, *n*(%) | 9 (19.1) | 51 (30.9) |
| Cumulative dose of MCT/LCT emulsion during W1, [*M* (*Q1,Q3*)], g/kg | 13.9 (10.4,16.0) | 12.8 (9.4,13.9) |
| Days to regain BW, [*M* (*Q1,Q3*)], days | 10.3 (8.1,12.4) | 9.8 (6.4,11.6) |
| Weight growth velocity, [*M* (*Q1,Q3*)], g/kg/day | 12.6 (10.2,14.8) | 15.1 (13.4,18.6) |
| ^a^A complete course of antenatal betamethasone consisted of two intramuscular 12-mg doses that were administered to mothers 24 h apart.  ^b^Data for diseases and interventions were collected before the occurrence of LO-NEC.  GA: gestational age; BW: birth weight; SGA: small for gestational age; LO-NEC: late-onset NEC; NRDS: neonatal respiratory distress syndrome; EOS: early-onset sepsis; hsPDA: hemodynamically significant patent ductus arteriosus; IMV: invasive mechanical ventilation；MCT/LCT: medium-chain and long- chain triglyceride. | | |
